# Time Course of Structural, Functional, Complement Changes and Inflammatory Processes in a Sodium Iodate Rat Model of Geographic Atrophy

**DOI:** 10.1096/fj.202502226R

**Published:** 2025-12-04

**Authors:** Shiying Zhao, Florian Voegele, Jiaqi Tang, Simon J. Clark, Alexander V. Tschulakow, Sylvie Julien

**Affiliations:** ^1^ Centre for Ophthalmology, University Medical Center Eberhard Karls University Tuebingen Germany; ^2^ Institute for Ophthalmic Research, University Medical Center Eberhard Karls University Tuebingen Germany; ^3^ Lydia Becker Institute of Immunology and Inflammation, Faculty of Biology, Medicine, and Health University of Manchester Manchester UK

## Abstract

Geographic atrophy (GA) is characterized by the loss of choriocapillaris, retinal pigment epithelium (RPE) and photoreceptors and is an advanced form of age‐related macular degeneration (AMD)–a leading cause of central vision loss in the elderly. The development of effective treatments has been hindered by the lack of reliable animal models that recapitulate the structural, functional, and molecular hallmarks of GA. In this study, we established and extensively characterized a sodium iodate (NaIO_3_)‐induced model of GA in pigmented Long Evans rats using a comprehensive set of in vivo and histological techniques. NaIO_3_ was administered intraperitoneally at 80 mg/kg to induce bilateral retinal degeneration. Morphological, functional, and ultrastructural changes were evaluated using scanning laser ophthalmoscopy (SLO), optical coherence tomography (OCT), electroretinography (ERG), light and electron microscopy, and immunohistochemistry at pre‐dose and 3, 7, and 14 days post‐injection. The model exhibited typical GA features including choriocapillaris loss, RPE degeneration, photoreceptor death, Bruch's membrane remodeling, and mitochondrial damage. Complement activation (C3, C5b‐9) and immune cell infiltration (Iba1, CD68) were observed, along with gliosis and RPE65 loss. ERG analysis revealed profound and persistent functional deficits. These findings demonstrate that the NaIO_3_ rat model robustly mimics the key pathological events in GA, particularly at 7 days post‐injection, making it a suitable model for preclinical evaluation of therapeutic interventions targeting choriocapillaris and RPE protection.

## Graphical abstract

A sodium iodate (NaIO_3_)‐induced geographic atrophy (GA) model in pigmented rats was established using in vivo and histological analyses. Morphological, ultrastructural changes, complement and inflammatory processes were evaluated at pre‐dose and 3, 7, and 14 days post‐NaIO_3_ injection. The model exhibited typical GA features including choriocapillaris loss, RPE degeneration, photoreceptor death, Bruch's membrane remodeling, and mitochondrial damage. Complement activation (C3, C5b‐9) and immune cell infiltration (Iba1, CD68) were observed, along with gliosis and RPE65 loss.

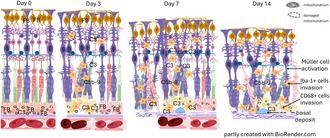

## 1 Introduction

Age‐related macular degeneration (AMD) is the leading cause of central vision loss in elderly individuals in developed countries. In 2020, approximately 200 million people were affected, and forecasts suggest that this number could rise to 300 million by 2040 [1]. In its early phase, AMD progresses slowly and presents in a non‐exudative form. This stage is characterized by progressive restructuring of the choriocapillaris and may advance to the rapidly progressive, exudative late‐stage form of AMD (wet AMD) or the non‐exudative late‐stage form, geographic atrophy (GA) (dry AMD). GA is defined by the loss of choriocapillaris (CC), retinal pigment epithelium (RPE) and photoreceptors. AMD is driven by a combination of genetic predisposition, natural aging processes and lifestyle factors, such as smoking and diet. The mechanisms through which these risk factors interact and contribute to disease progression remain poorly understood, making drug discovery particularly challenging. To date, no therapeutic approach has proven fully effective. However, genetic and molecular studies have identified the complement system as an important player in AMD pathogenesis [2]. Until 2023, there was no approved treatment for the advanced form of dry AMD with GA. Now, and only in the United States, two complement protein inhibitors: Pegcetacoplan and avacincaptad pegol (targeting C3 and C5 respectively) have been approved by the United States Food and Drug Administration (FDA) as treatments for GA. Both slow down the progression of GA secondary to AMD by modulating the complement system [3]. Since Pegcetacoplan was approved by the FDA in the USA as the first drug against GA in spring 2023, ophthalmologists and many patients expected an approval by the European Medicines Agency (EMA) in Europe in 2024. Unfortunately, the EMA recommended refusing marketing authorization for Pegcetacoplan. The EMA's Committee for Medicinal Products for Human Use (CHMP) justified the negative vote as follows: the committee acknowledged that the drug effectively slowed the morphological progression of GA lesions—but noted that this did not translate into a clinically meaningful benefit in terms of visual function or quality of life during the study period. Furthermore, the CHMP emphasized the risks associated with repeated intravitreal injections, including conversion to wet AMD or and intraocular inflammation, which could further worsen vision. The agency therefore concluded that the benefits of Pegcetacoplan did not outweigh the risks and the approval was refused.

A major barrier to the development of effective treatments for GA has been the lack of optimal and well‐characterized animal models. Several experimental models have been established to mimic GA‐like conditions in animals, including knockout models, laser models, oxidative stress models, A2E accumulation models, light‐toxicity models, natural aging models and others more [4]. Among these, the sodium iodate (NaIO_3_) model is a particularly promising candidate. It closely mimics GA‐associated pathology, including Bruch's membrane remodeling, gliosis and immune activation, progressive loss of CC, RPE, and photoreceptors.

The NaIO_3_ model has been established in numerous animal species including rats, rabbits, cats, primates, pigs and frogs [5] to investigate retinal degeneration. However, the degeneration induced by NaIO_3_ is highly dependent on time and dosage, as well as dependent on the animal species and the route of administration (intravenous, intraperitoneal, subretinal, intravitreal or retroorbital) [6, 7]. Therefore, there is a clear need for a thoroughly documented NaIO_3_ model that enables detailed analysis of the structural and temporal progression of GA‐related degeneration in key tissues, including the CC, RPE and photoreceptors, prior to testing therapeutic interventions. In this study, pigmented rats were selected because of the known property of certain drugs to specifically bind to melanin pigments and remain in pigment cells for long periods of time. Therefore, this potential melanin binding can significantly influence drug retention in ocular tissues and thus affect treatment outcomes [8]. Furthermore, it has recently been demonstrated that complement activation, particularly via the classical pathway is more similar between rats and humans than between mice and humans [9].

NaIO_3_ was administered systemically to induce reproducible GA‐like pathology in both eyes of the animals. Structural and functional alterations were assessed over a two‐week period using in vivo imaging, electroretinography (ERG) and histology. Ultrastructural analyses were performed using electron microscopy, to examine CC, Bruch's membrane, RPE and photoreceptors in detail for morphological alterations. In addition, local complement activity and inflammatory processes related to GA development were evaluated using immunohistochemistry. Our model provides a valuable platform for evaluating new therapeutic strategies aimed at preserving the CC and RPE and preventing the development of GA in the context of AMD.

## 2 Materials and Methods

### 2.1 Ethics Statement

This study involved experiments conducted on rats. All procedures were approved by the Animal Experimentation Committee of the University of Tuebingen “Regierungspraesidium Tuebingen” (AK 01/21G). All animals were handled in accordance with the German Animal Welfare Act and were under the supervision of the university's animal protection agency and attending veterinarians at the Eberhard‐Karls University of Tuebingen. No human experiments were involved in this study.

### 2.2 Animals and Experimental Design

Six‐week‐old female Long Evans (LE) rats were purchased from Janvier Labs (Le Genest‐Saint‐Isle, France). Female rats were chosen due to their docile behavior. A total of 12 rats were used in this study. Rats received intraperitoneal (i.p.) injections of NaIO_3_ (80 mg/kg body weight). Untreated rats served as controls (day 0, pretreatment). Groups of three animals (six eyes) were examined in vivo using electroretinography (ERG) and scanning laser ophthalmoscopy/optical coherence tomography (SLO/OCT) at 0 (pretreatment), 3, 7 and 14 days post‐ injection. Then, the eyes were enucleated and examined histologically. One eye was used for light and electron microscopy (in total three eyes from three rats) and the contralateral eye for immunohistochemistry (three eyes from three rats).

### 2.3 Electroretinography (ERG)

Animals were dark adapted overnight prior to ERG measurements. The rats were anesthetized by an intraperitoneal injection of three‐component narcosis (0.005 mg fentanyl, 2 mg midazolam and 0.15 mg of medetomidine/kg body weight). Pupils were dilated with 1 to 2 drops of a Mydriaticum (Pharmacy of the University of Tuebingen, Germany); additionally the topical anesthetic Novesine (OmniVision, Puchheim, Germany) was applied. Gold ring electrodes (1.5 mm diameter), (Roland Consult, Stasche & Finger GmbH, Brandenburg, Germany) were placed on the corneas of both eyes. Methocel eye drops (OmniVision, Puchheim, Germany) were used to ensure contact and prevent corneal drying. Subdermal platinum (27 gauge) needles (Technomed Europe, Maastricht, The Netherlands) in the forehead between the eyes and at the base of the tail served as reference and ground electrodes, respectively. Light stimuli were delivered in a Ganzfeld dome (Roland Consult, Brandenburg, Germany). ERG‐response amplitudes were measured using a standard protocol. Dark‐adapted, responses were elicited by brief flashes of white light on a dark background. Two stimulus intensities were used: a low one (0.01 cd s/m^2^) to analyze the rod function and a high one (10 cd s/m^2^) to analyze the mixed rod‐cone function. The RETIsystem software (Roland Consult, Brandenburg, Germany) was used for recording and analysis of the ERG data. A system‐internal 50 Hz filter was used for background suppression. After that, the a‐ wave and b‐wave amplitudes were determined from the electroretinograms.

### 2.4 In Vivo Imaging (SLO/OCT)

Immediately following the ERG analysis, SLO and OCT imaging were performed under continued anesthesia. The pupils were repeatedly dilated with 1 to 2 drops of Medriaticum (Pharmacy of the University of Tuebingen, Germany) and an additional drop of the topical anesthetic Novesine (OmniVision, Puchheim, Germany) was applied. Methocel (OmniVision, Puchheim, Germany) eye drops were used to prevent drying of the eyes. SLO and OCT were performed using a Spectralis HRA + OCT device (Heidelberg Engineering, Heidelberg, Germany). To adapt it for analyses in rats a +78D double aspheric lens (Volk Optical Inc., Mentor, OH 44060, USA) was placed directly on the outlet of the device. An additional custom‐made +7 diopter (dpt) contact lens was placed directly on the rat's eyes. For the proper positioning during the in vivo examination, the rats were put on an adjustable platform in front of the device. The near‐infrared reflectance (NIR‐R) mode was used first to align the camera and acquire well‐focused images centered on the optic nerve head (ONH). Then, the fundus autofluorescence was recorded in the short‐wavelength autofluorescence (SW‐AF) mode. The fundus fluorescence images were recorded using a 55° camera angle. Twenty consecutive frames were captured to generate an averaged image. OCT scans were then manually acquired in the central ONH region. For the measurement of the mean retinal thickness on these images, a semi‐automatic ImageJ macro‐based approach was used (InteredgeDistance_v1.4_ImageJMacro).

### 2.5 Light Microscopy (LM) and Transmission Electron Microscopy (TEM)

For histological analysis, the rats were sacrificed directly after in vivo examinations at the designated time points. One eye per animal was fixed in 5% glutaraldehyde overnight at 4°C and then embedded for ultra‐thin sectioning following standard procedures. Briefly, the eyes were postfixed with 1% osmium tetroxide in 0.1 M cacodylate buffer (pH 7.4), followed by staining with saturated uranyl acetate and dehydration with a graded series of ethanol and propylene oxide. The specimens were then embedded in Epon. Reagents were obtained from Serva (Heidelberg, Germany), AppliChem (Darmstadt, Germany) and Merck (Darmstadt, Germany). Semi‐thin sections (700 nm) stained with toluidine blue were investigated by light microscopy (Zeiss Axioplan2 imaging system; Zeiss, Jena, Germany). Ultra‐thin sections (70 nm) were examined by electron microscopy (Zeiss EM 900; Jena, Germany).

### 2.6 Immunohistochemistry (IHC)

The contralateral eye of each animal was fixed in 4.5% paraformaldehyde (Carl Roth, Karlsruhe, Germany) for three days and embedded in paraffin using standard procedures. The eyes were cut in 4‐μm thick sections. For the IHC staining sections adjacent to the ONH were used. The staining procedures were performed following the instructions provided by the manufacturer. Primary antibodies against RPE65 (1:500; Thermo Fisher SCIENTIFIC, Germany), Iba1 (1:1000; FUJIFILM Wako, Japan), GFAP (1:100; Cell Signaling Technology, USA), vimentin (1:100; Bio‐Techne, Germany), CD68 (1:100; Bio‐Rad, Germany), C3 (1:2000; abcam, Germany), C5 (1:500; NSJ Bioreagents, USA), C5b‐9 (1:500; biorbyt, UK), FB (1:100; abcam, Germany) and FH (1:100; BIOSS ANTIBODIES, USA) were used. Secondary antibodies (Jackson ImmunoResearch, UK) were applied accordingly and 4′,6‐Diamidino‐2‐phenylindole (DAPI) was used to counterstain the nuclei. Slides were cover‐slipped with fluorescence mounting medium (Dako Omnis, Denmark) and analyzed using a Zeiss Axioplan2 imaging microscope (Zeiss, Jena, Germany). Adjacent sections were stained with hematoxylin and eosin (HE) for additional morphological evaluation.

### 2.7 Quantification of Photoreceptor Nuclei

For the analysis both semithin and hematoxylin and eosin (HE) stained paraffin sections were used. Overlapping images from each section were recorded at a magnification of 630× using the Zeiss Axioplan2 imaging system. These single images were used to create multi‐image alignments (MIA). On these MIAs, the number of nuclei was quantified within each 50‐μm interval starting next to the ONH and continuing superiorly and inferiorly along the vertical meridian. The area from 1 mm superior to 1 mm inferior from the ONH was included in the analysis (Figure 2A).

### 2.8 Quantification of Mitochondria in the RPE on Electron Micrographs

For each group, 10 randomly chosen RPE‐containing electron micrographs with a magnification of 12 000× were analyzed. The total number and the number of damaged mitochondria in the RPE cells were counted manually.

### 2.9 Statistics

All statistical analyses were performed using Prism 8.0 (GraphPad Software, USA). The results are presented as mean ± standard deviation (SD). For statistical analyses involving multiple groups, ANOVA for multiple comparisons was utilized. Linear regression analysis was conducted using the corresponding “linear regression analysis”‐function within the software.

## 3 Results

### 3.1 Follow‐Up of Retinal Degeneration Using In Vivo Imaging and Light Microscopy

**FIGURE 1:**
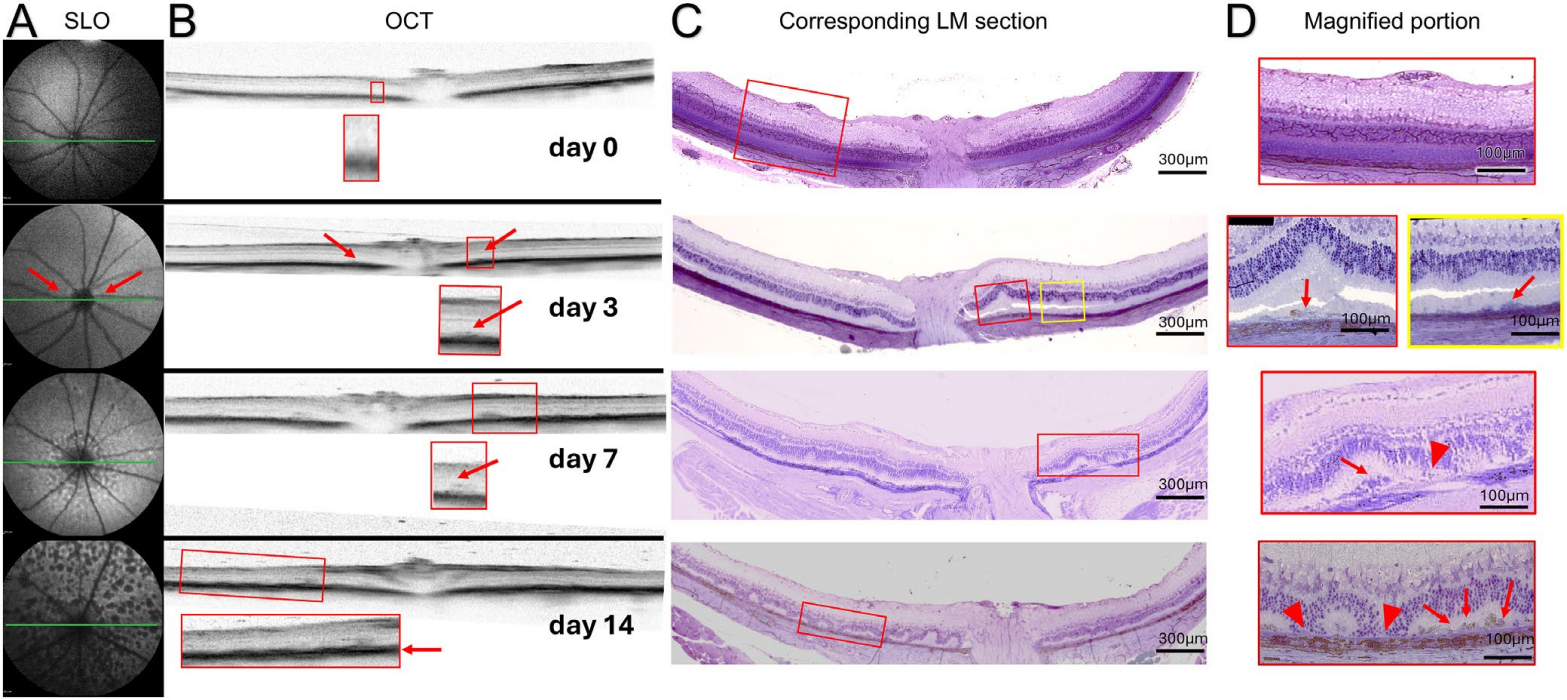
Time course of NaIO_3_‐induced retinal degeneration using in vivo analysis and light microscopy (LM): (A) SLO‐fundus auto‐fluorescence images of an untreated control eye (day 0) and eyes 3, 7 and 14 days after NaIO_3_ injection. Red arrows point at a hyperfluorescent ring around the optic nerve at day 3. (B) Corresponding OCT, the cross‐sections at the level of the green lines in (A) are shown. The red arrows point at areas with hyperreflective changes. (C) Corresponding LM sections. (D) Magnified portions of the red and yellow boxes shown in (C). The red arrows indicate areas with RPE agglomerations, the red arrowheads indicate areas with total loss of photoreceptor outer segments (OS). At the time points 3‐ and 7‐days post NaIO_3_ injection, no changes can be observed in the periphery, whereas at day 14 the changes span over the whole area of observation.

On day 0 (pretreatment) the fundus auto‐fluorescence of the back of the eye was homogeneous, as observed in SLO (Figure 1A). The corresponding OCT (Figure 1B) and LM (Figure 1C,D) images showed no abnormalities.

On day 3, a hyper‐autofluorescent ring had appeared around the optic nerve in SLO (Figure 1A). On the OCT image (Figure 1B) in the corresponding area, hill‐like structures in the RPE reflection area emerged (indicated by red arrows). The corresponding LM images revealed RPE cell agglomeration and the onset of rosette formations in these areas (Figure 1C). Additionally, as exemplified by the yellow box in Figure 1D, morphological changes and migration of the RPE cells were noted outside the areas that showed abnormalities in the in vivo imaging.

On day 7, SLO images displayed a pattern of hyper‐ and hypo‐ auto‐fluorescent areas in the back of the eyes (Figure 1A). OCT revealed wave‐like distortions in the, RPE reflection area. Significant retinal thinning was measured (Figure S2). LM analysis showed alternating regions of RPE agglomeration and RPE loss (Figure 1D red arrows). Rosette formations were present throughout the retina, with areas of partial and total loss of photoreceptor outer segments (red arrowheads).

On day 14, in the SLO the hyper‐ and hypo‐autofluorescent areas became larger and more distinct. In the corresponding OCT, the RPE reflection area changes became more pronounced compared to day 7 and a further retinal thinning was measured (Figure S2). The LM images revealed enhanced rosette formation, with areas of partial and total loss of outer segments of photoreceptors (red arrowhead) and areas of RPE agglomeration (Figure 1D red arrows).

### 3.2 Quantification of Photoreceptor Nuclei Loss Over Time After NaIO_3_ Injection

**FIGURE 2:**
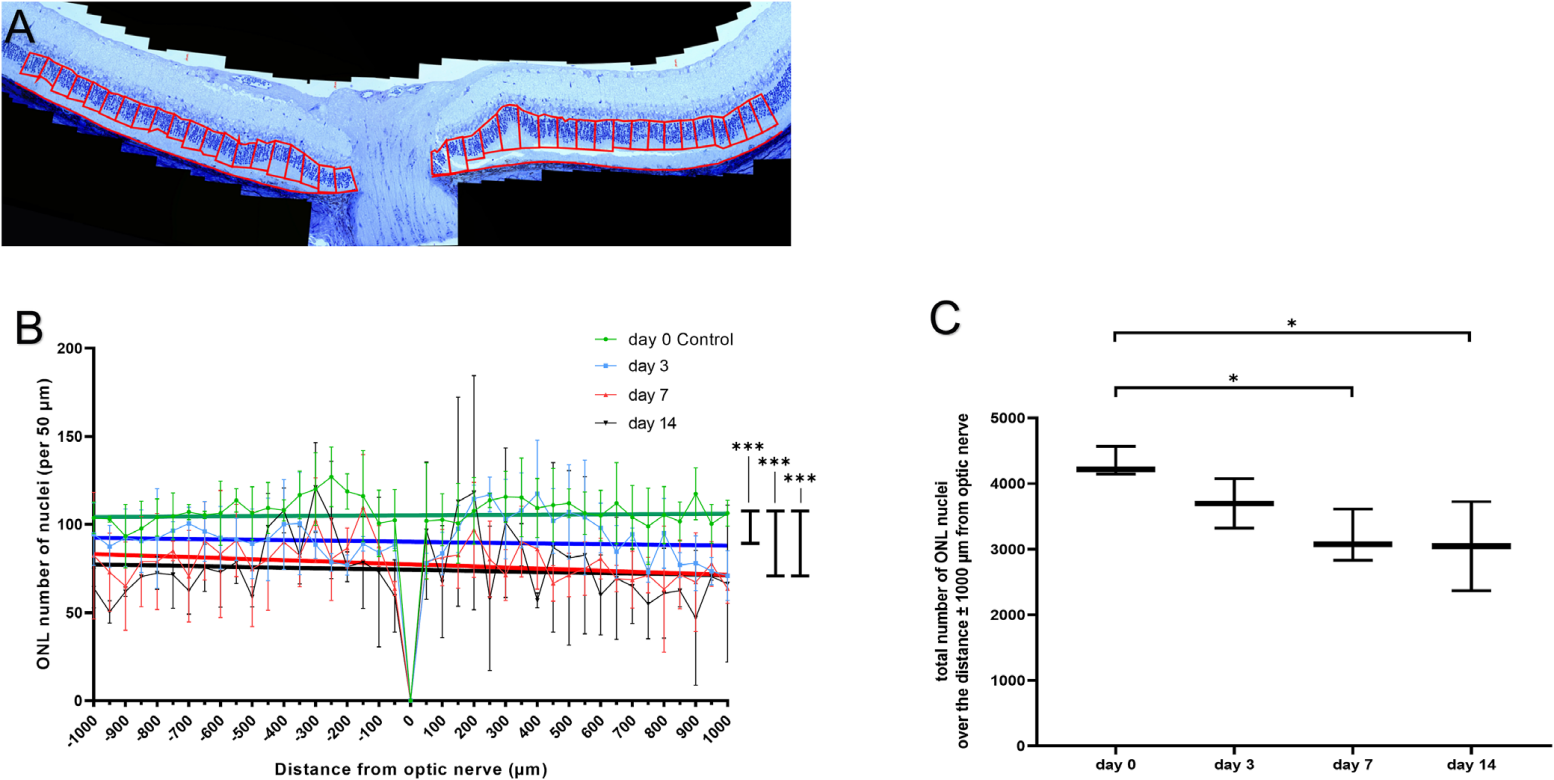
Quantification of photoreceptor nuclei for the different analysis time points after NaIO_3_ injection: (A) principle of the analysis: the ONL nuclei were counted for each 50 μm retinal length over a length of 1000 μm to each side of the optic nerve. (B) the results for the quantification for each 50 μm retinal length for the untreated group in green, 3, 7 and 14 days after NaIO_3_ injection in blue, red and black respectively. The linear correlation lines for each group are each shown in the corresponding color. The analysis of the elevation and intercepts of the linear regression showed highly significant result ***p < 0.0001. (C) Statistical analysis of the total numbers of the ONL nuclei for each group: 0 days (pretreatment), 3, 7 and 14 days after NaIO_3_ treatment, *n* = 3 eyes per group ANOVA with Dunn‘s posttest, *p < 0.05.

Photoreceptor nuclei were counted per 50‐μm retinal length to quantify the process of degeneration (Figure 2A). A gradual reduction of photoreceptor nuclei was observed over time. Linear regression analysis showed that the elevations and intercepts for 3, 7 and 14 days post NaIO_3_ injection were significantly lower compared to the results of the untreated control (Figure 2B). Total nuclei counts were significantly lower at 7 and 14 days after NaIO_3_ injection compared to the control (day 0, no NaIO_3_ injection) (Figure 2C).

### 3.3 Ultrastructural Changes Induced by NaIO_3_ Over Time

#### 3.3.1 Ultrastructural Changes in the Retina/RPE/CC/Choroid

**FIGURE 3:**
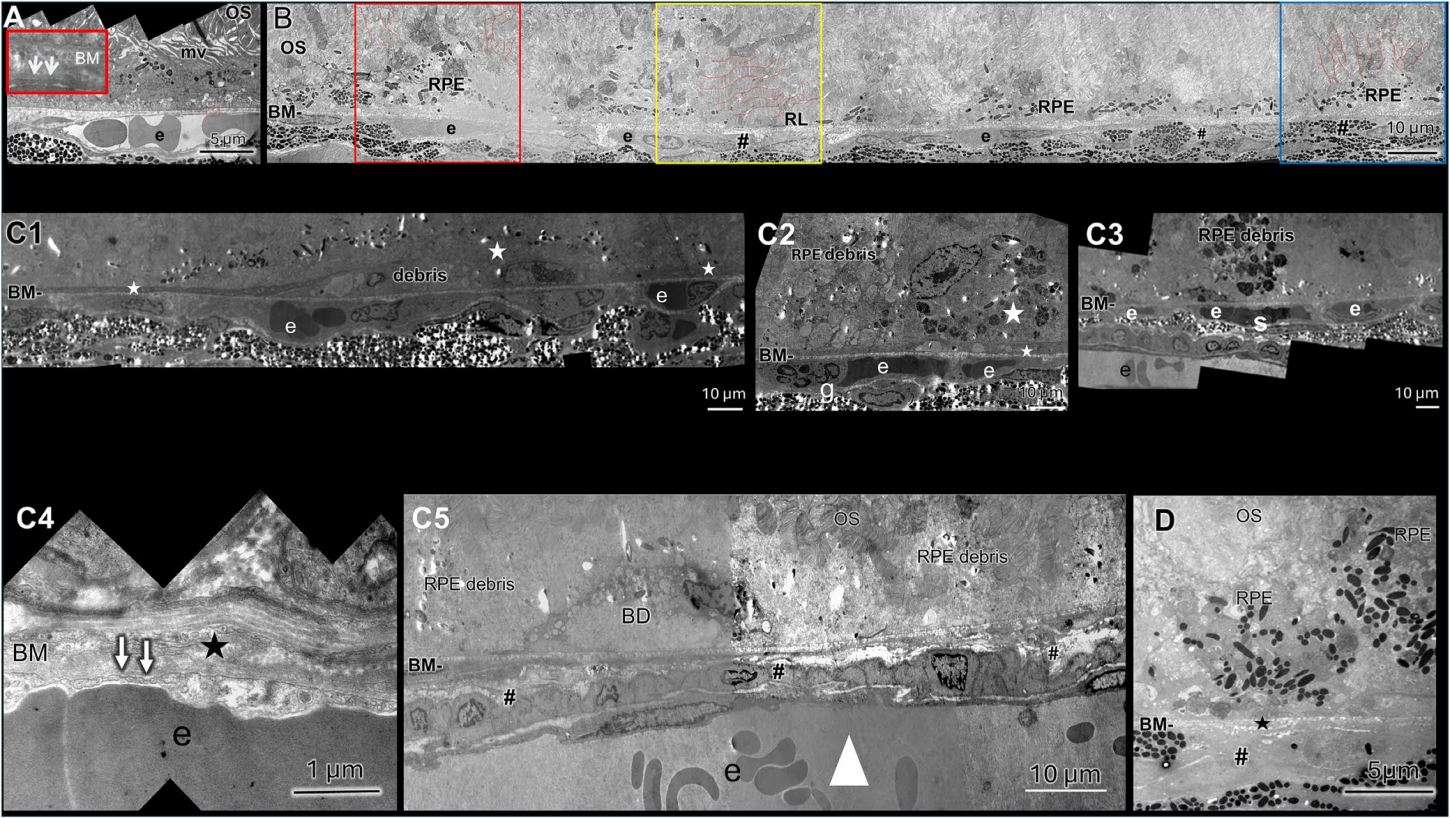
Ultrastructural changes induced by NaIO_3_ over time: (A) electron micrograph of GA key players that is, retina/RPE/CC/choroid in a healthy state (untreated control). CC with erythrocytes (e) and open lumen, healthy RPE with pigments, basal labyrinth and microvilli (mv), photoreceptor outer segments (OS) perpendicular to the Bruch's membrane. The red box (shown as a magnified image in the upper left corner) shows a normally organized Bruch's membrane (BM) and the choriocapillaris vessel wall with fenestrations indicated by white arrows. (B) Electron micrograph showing the ultrastructural changes 3 days after NaIO_3_ injection. (Red box) CC vessel with reduced lumen and an erythrocyte (e) and overlying RPE cells at an early stage of multilayer‐formation with a changed morphology, flattened appearance, no microvilli (mv). The majority of the surrounding OS (indicated in red) with disheveled organization but perpendicular orientation to the Bruch's membrane. (Yellow box) area with no CC (#), no RPE (RL) and a changed direction of OS (indicated in red). (Blue box) area with CC loss (#), but overlying RPE cells with a changed morphology. The majority of the OS showed a perpendicular orientation to the Bruch's membrane (indicated in red). (C1–C5) electron micrographs illustrating different ultrastructural changes observed on day 7 after NaIO_3_ injection. (C1) and (C2) Strong evidence of inflammation. (C1) Long microglia cell or macrophage lying on the BM filled with debris (indicated by white stars ✰). (C2) CC with a granulocyte (g) next to erythrocytes (e). On the BM, the smaller white star (✰) marks a thin microglia cell or microphage with an overlying microglia cell or microphage filled with pigmented debris (larger white star ✰). (C3) Area with degenerating RPE (RPE debris) over CC vessels, the central vessel shows an accumulation of erythrocytes indicating stasis (s). (C4) BM with a changed composition of its (ECM) (★) and deposits of abnormal material, CC with fenestrations indicated by white arrows. (C5) Basal deposit (BD) between the BM and the RPE debris, large choroidal vessel unusually close to the BM (white arrowhead) extended region with CC loss (#). (D) Electron micrograph showing the typical ultrastructural changes observed 14 days after NaIO_3_ injection: CC loss (#), melanocytes directly touching the BM, a thickened BM with changed composition of the ECM indicated by the black star (★), degenerated RPE cells and damaged OS.

Three days post NaIO_3_ injection, severe ultrastructural changes have been observed (Figure 3B). The organization and orientation of the OS appeared disheveled and in some areas the OS were oriented not perpendicular to Bruch's membrane (yellow box). Alternating regions of RPE agglomeration and RPE loss have been observed. The RPE cells showed changes in morphology like reduced thickness and loss of intercellular junctions and microvilli (red and blue boxes). The CC vessels often had reduced vessel lumens (red box). Extensive areas with CC loss have been observed, in particular, regions with CC loss and still present RPE cells (blue box) and regions lacking both CC and overlying RPE (yellow box). Interestingly, no regions were found where the CC was present without overlying RPE.

At day 7 post NaIO_3_ injection, in addition to pronounced degenerative changes of RPE and photoreceptors (Figure 3C1–C3,C5), strong evidence of inflammatory processes at the CC‐RPE interface was observed. Numerous microglia cells or macrophages filled with debris appeared (Figure 3C1,C2), and granulocytes were detected within the CC (Figure 3C2). The majority of the CC vessels exhibited reduced lumens (Figure 3C1–C3). In some vessels accumulations of stuck erythrocytes were observed, indicating stasis (Figure 3C3). The composition of the extracellular matrix (ECM) of the Bruch's membrane was thickened and disheveled and contained deposits of abnormal material (Figure 3C4). Basal deposits over the Bruch's membrane, similar to those found in AMD patients appeared. Compared to day three, the areas with CC loss became more extensive; additionally frequently large choroidal vessels were observed unusually close to the Bruch's membrane (Figure 3C5).

Fourteen days after NaIO_3_ injection the ultrastructural changes were even more pronounced, but remained similar to those, observed at seven days post‐injection (Figure 3D).

#### 3.3.2 Mitochondrial Morphological Changes Induced by NaIO_3_ Over Time

**FIGURE 4:**
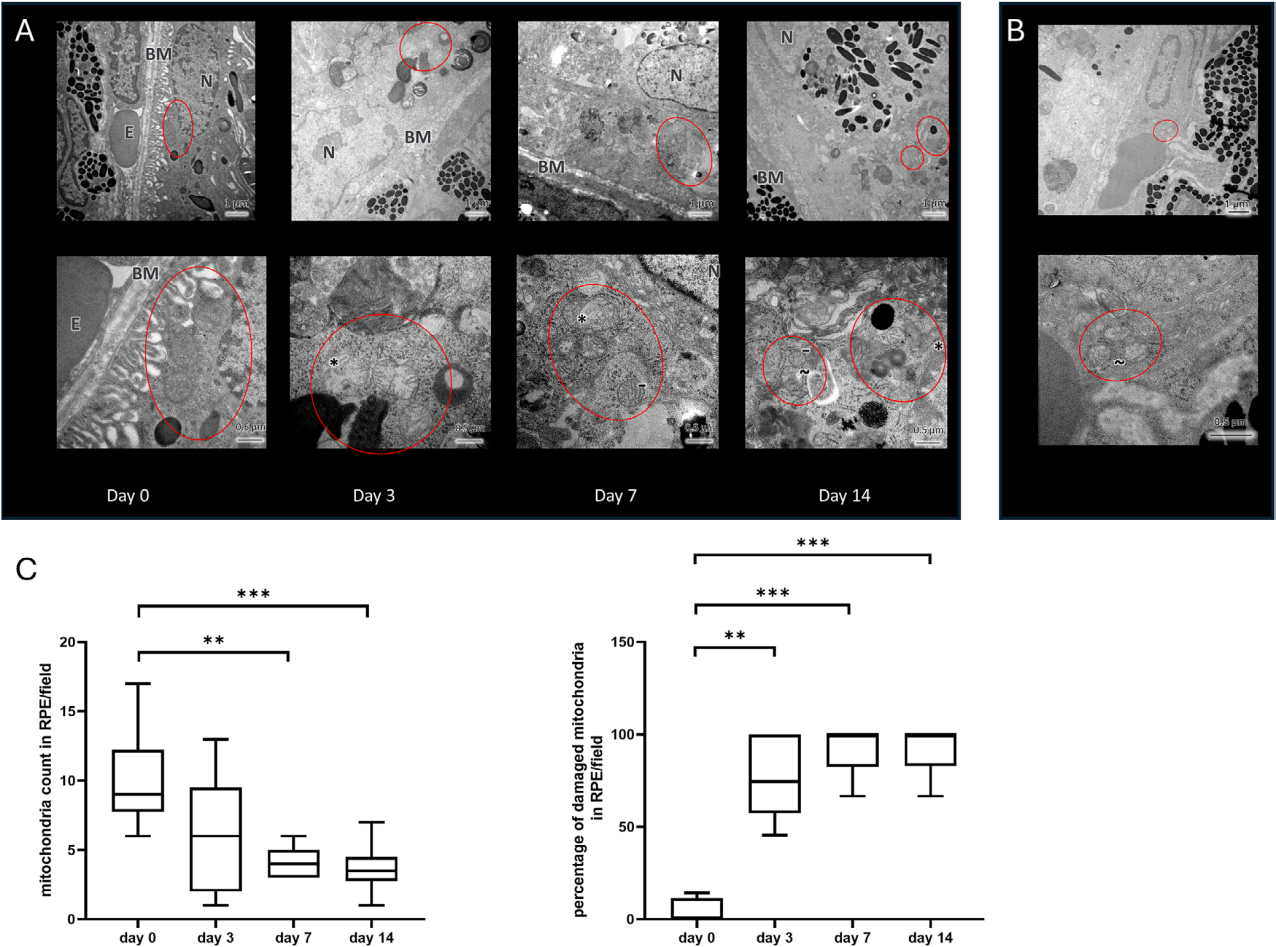
(A) RPE mitochondria at different time points after NaIO_3_ injection (untreated control day (0), day 3, day 7 and day 14). The upper images (7000× magnification) show mitochondria containing RPE areas (circled in red). The lower images show the same areas under 20 000× magnification. Bruch's membrane (BM), erythrocyte (E), nucleus (N). Day 0: Three healthy mitochondria (circled in red) with intact membranes and cristae. Day 3: Three mitochondria displaying characteristic morphology changes observed after oxidative stress, like reduction of mitochondrial inner membrane folds (cristae), swelling, mitochondrium with ruptured membrane (*). Day 7: Mitochondria at different stages of damage (circled in red), displaying similar characteristics as described for day 3, a shrunk mitochondrium is marked with (−). Day 14: Mitochondria at different stages of damage (circled in red), displaying similar characteristics as described for day 3 and 7, a mitochondrium with blebbing surface is marked with (~). (B) Mitochondrial damage in an endothelial cell of the choriocapillaris on day three after NaIO_3_ injection. Similar to the mitochondria in the RPE, here the mitochondria (circled in red) exhibited characteristic morphology changes observed after oxidative stress, like reduction of mitochondrial inner membrane folds (cristae), deformation, membrane blebbing (~). The endothelial cell itself appeared swollen. (C) Statistical analysis of the number of mitochondria in the RPE (left panel) and the percentage of damaged mitochondria (right panel) per microscopic field (10 fields/group, magnification 12 000×) *n* = 3 eyes per group, ANOVA with Dunn's posttest, **p < 0.001, ***p < 0.0001.

Three days after NaIO_3_ injection, the mitochondria in the RPE and in endothelial cells exhibited characteristic morphological changes observed after oxidative stress, such as reduction of mitochondrial inner membrane folds (cristae), changes in the form and structure, swelling or shrinking, bleb formation and ruptures of the membranes (Figure 4A,B). Even more damaged mitochondria were observed 7 and 14 d after NaIO_3_ injection (Figure 4A). The total number of mitochondria in the RPE was significantly reduced seven and 14 days after NaIO_3_ injection compared to the levels at day 0 (Figure 4C left panel), while the percentage of damaged mitochondria significantly increased three, seven and 14 days after NaIO_3_ injection compared to the day 0 results (Figure 4C right panel).

### 3.4 Inflammatory Changes Induced by NaIO_3_ Over Time

**FIGURE 5:**
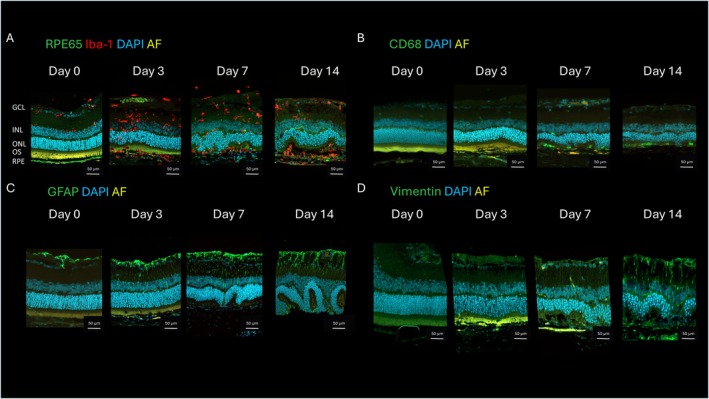
Immunofluorescence stainings showing inflammatory changes induced by NaIO_3_ over time. Nuclei were counterstained with DAPI (blue) and autofluorescence (AF) appeared in greenish yellow. (A) RPE65 signal (green) disappeared gradually with time indicating a gradual degeneration of the RPE. Iba1 signal (red) showed microglia or macrophage infiltration into the retina at sites of retinal degeneration. (B) CD68 (green) staining for macrophages. No macrophage is present on day 0, a few choroidal macrophages are visible on day 3, and a clear invasion of macrophages in the subretinal space is seen on days 7 and 14. (C) GFAP staining (green) for astrocytes and activated Müller cells. On day 0, only astrocytes can be seen in the inner neural retina. An activation of Müller cells can be observed starting at day 3 and increased up to day 14 as indicated by an enhancement of GFAP signal in the ganglion cell layer. (D) Vimentin staining (green) for Müller cells also showing their increasing activation starting at day 3.

To evaluate the effect of intraperitoneally applied NaIO_3_ on inflammatory changes in the retina, we performed immunohistochemical stainings of microglia/macrophages (Iba1) (Figure 5A), phagocyting macrophages (CD68) (Figure 5B) and Müller cells (GFAP, vimentin) (Figure 5C,D). Additionally, the expression of RPE65 was analyzed (Figure 5A). We found an invasion of Iba1^+^ microglia/macrophages within the retina and subretinal Iba1^+^ cells associated with OS already three days after NaIO_3_ treatment as well as after seven and 14 days with similar intensity (Figure 5A). Our staining revealed a progressive RPE65 signal loss from day 3 post‐injection onwards (Figure 5A). Only a few choroidal CD68^+^ macrophages were visible three days after NaIO_3_ treatment, which was followed by a subretinal invasion of CD68^+^ macrophages after seven and 14 days (Figure 5B). Increasing activation of Müller cells that is, Müller cell gliosis was observed during the investigated period after NaIO_3_ treatment, indicated by an increasing GFAP and vimentin signal intensity (Figure 5C,D).

### 3.5 Complement Proteins Changes Induced by NaIO_3_ Over Time

**FIGURE 6:**
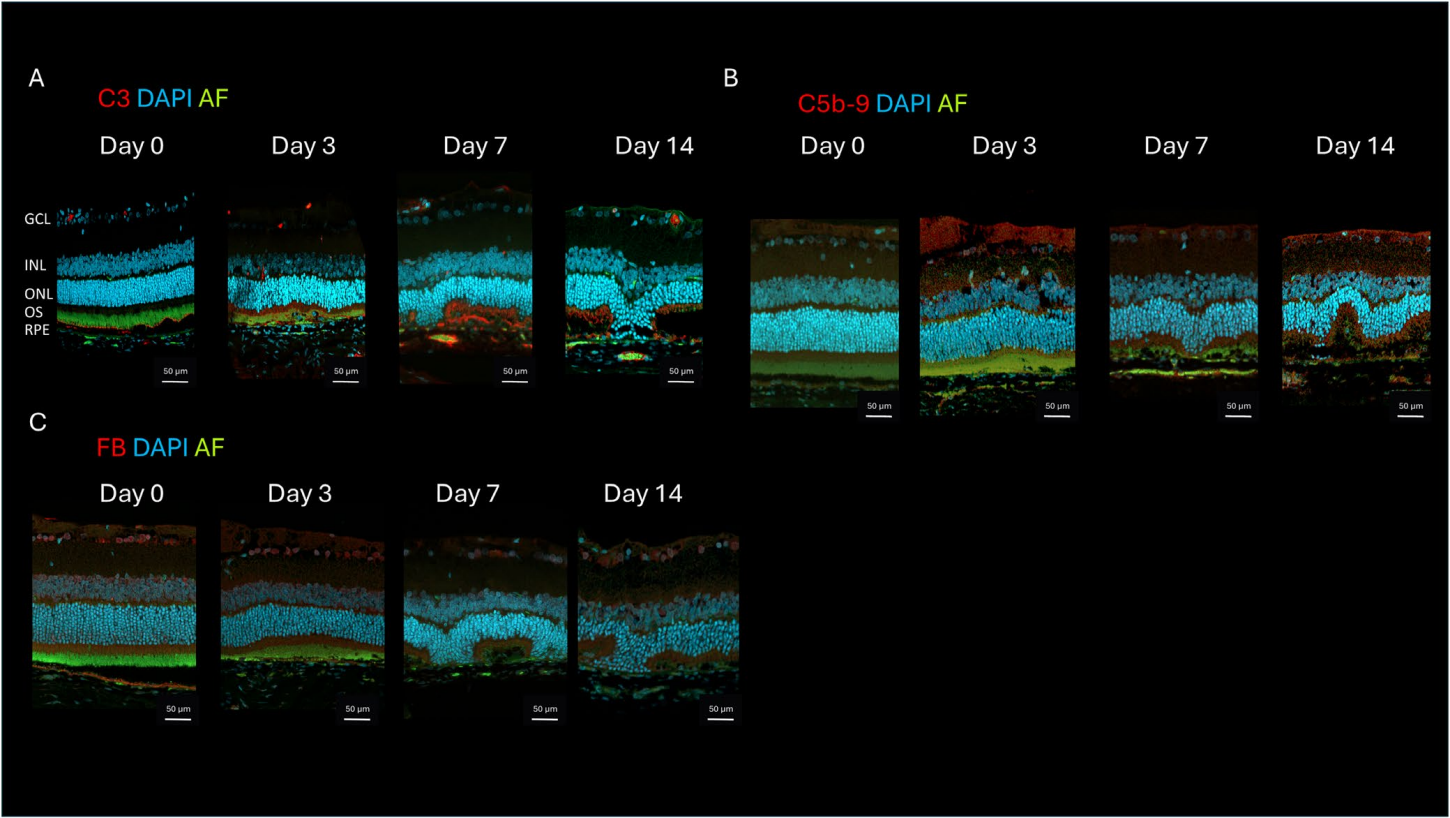
Immunohistochemical stainings showing complement changes induced by NaIO_3_ over time. Nuclei were counterstained with DAPI (blue) and autofluorescence (AF) appeared in greenish yellow. (A) On day 0, the complement factor C3 signal (red) is mainly located at the RPE close site of the OS of the photoreceptors, three days after NaIO_3_ injection the signal started to increase in intensity peaking on day 7 and then the signal slightly decreased at day 14. (B) No C5b‐9 signal (red) was detected on day 0. On days 3, 7 and 14, a signal was detected in the inner neural retina. The signal intensity peaked in the OS and RPE/CC region at the time points 7 and 14 days post‐injection. (C) On day 0, Factor B (FB) immunoreactivity (red) was localized in the RPE and the inner neural retina region and slightly decreased over time.

To further understand the molecular mechanisms underlying oxidative stress‐induced retinal degeneration, we performed immunohistochemical stainings of C3, C5b‐9, FB, C5 and FH. The staining revealed increased C3 protein accumulation in the OS after NaIO_3_ treatment with a peak at day seven. Fourteen days after NaIO_3_ treatment, a lower staining was still detectable which correlated with the OS degeneration (Figure 6A). Additionally, C5b‐9 protein accumulation was detected in the inner neural retina on days 3, 7 and 14, compared to control animals (Figure 6B), which provided direct evidence of NaIO_3_‐induced complement activation in rat retinal tissues. In contrast, factor B (FB) immunoreactivity was localized in the RPE and the inner neural retina only on day 0, and decreased over time (Figure 6C). No C5 or FH staining was detected at any of the analyzed time points (Figure S1).

### 3.6 In Vivo Assessment of the Retinal Function Using Electroretinography (ERG)

**FIGURE 7:**
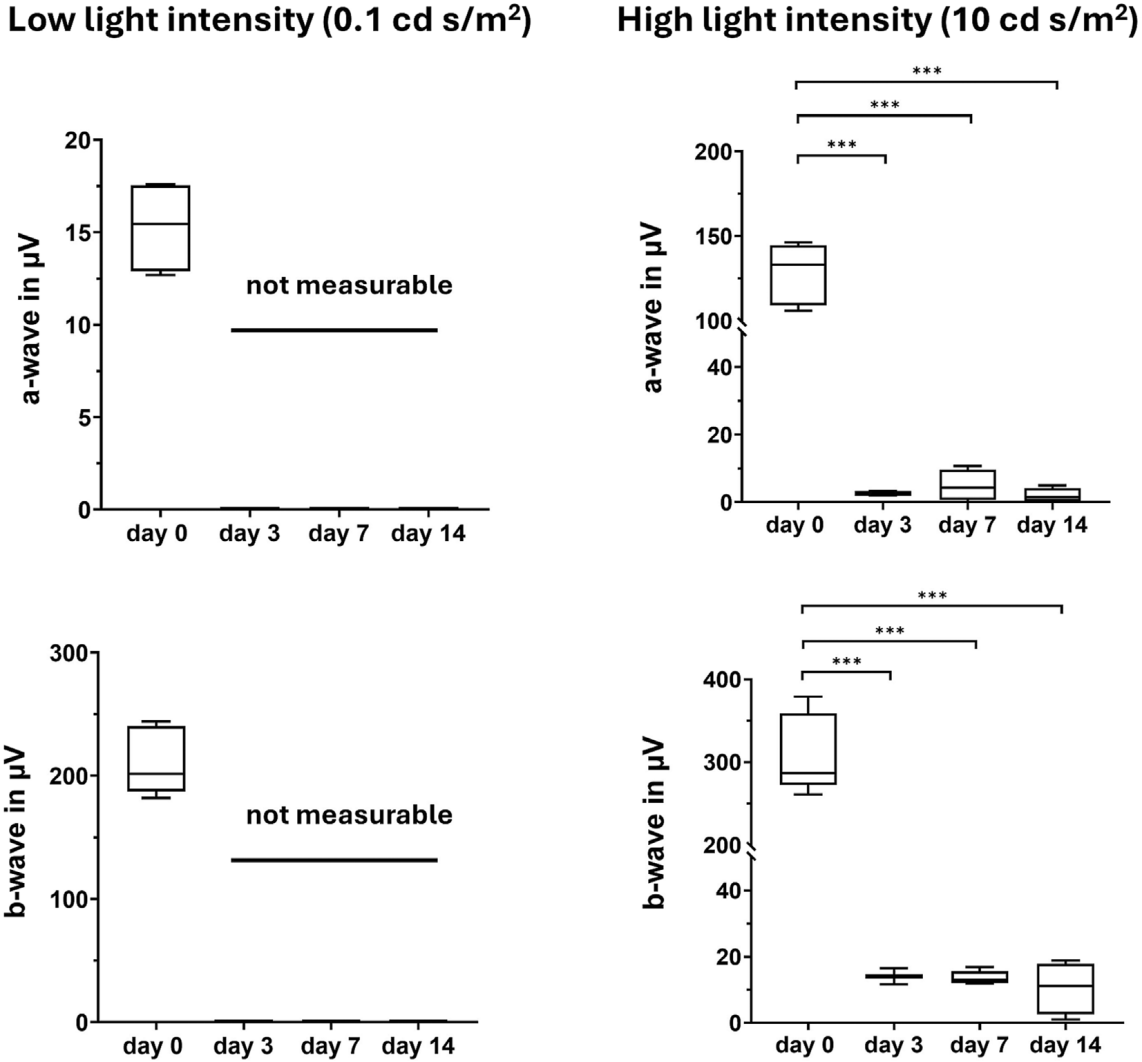
Analysis of the a‐ and b‐wave amplitudes in rats before NaIO_3_ treatment (d0) and 3, 7 and 14 days after treatment for two stimulus intensities: low (0.1 cd s/m^2^) and high (10 cd s/m^2^). *n* = 6 eyes per group, ANOVA with Dunn's post test, ***p < 0.0001.

The a‐ and b‐wave amplitudes after low light intensity flashes (0.1 cd s/m^2^) were not measurable at any of the time points after NaIO_3_ injection. The a‐ and b‐wave amplitudes measured after high light intensity flashes (10 cd s/m^2^) were severely reduced already at the time point of three days after NaIO_3_ injection. Similar results have been obtained seven and fourteen days after NaIO_3_ injection (Figure 7).

## 4 Discussion

The aim of this study was to establish and thoroughly characterize a simple, effective, and rapid NaIO_3_‐induced pigmented rat model that closely replicates the key symptoms of GA for preclinical testing of treatment approaches. To achieve a detailed characterization, we employed a broad spectrum of in vivo and histological analyses, including SLO/OCT, light and electron microscopy, IHC and importantly, given the evolving regulatory environment regarding future therapies, we included functional testing via ERG.

We used pigmented rats because certain drugs are known to bind to melanin. Thus, our model allows for the simulation of potential melanin‐related drug binding in future ocular drug testing.

Systemic ip injections were chosen to avoid complications such as trauma‐induced cataract, damage to target tissues, and increased inter‐individual variation, which are commonly associated with intravitreal or subretinal injections. The systemic application resulted in highly similar effects in both eyes, allowing one eye to serve as an internal control in preclinical experiments.

A dosage of 80 mg/kg NaIO_3_ was selected to induce reliable, non‐reversible effects, simulating the patient situation. It was shown that lower doses (e.g., 20 mg/kg) lead to reversible effects on retinal tissues [10] or have no measurable effects [11], depending on the animal model and injection route used. Higher doses can be lethal. Animals were analyzed at 3, 7, and 14 days post NaIO_3_ injection.

The most prominent NaIO_3_‐ induced early changes observed on day 3 post‐injection were located in a ring‐like area approximately 300 μm from the optic nerve head. These changes led to the appearance of a hyper‐fluorescent ring in SLO and a thickening of the RPE reflection area on the corresponding OCT images (Figure 1A,B respectively). The LM images of these regions showed a strong accumulation of highly pigmented RPE cells and initial rosette formation directly above these areas (Figure 1C,D). These changes appear unique to LE rats, as no equivalent findings have been reported in other rat strains or in mice, neither at 3 days post‐injection, nor at comparable, or later time points. In mice, three days post NaIO_3_ injection, areas showing changes in SLO‐AF and OCT reflectivity were located in a broader area around the ONH, being closer to our findings 7 days post‐injection [12, 13].

Additionally, LM revealed morphological changes and migration of RPE cells outside the areas which showed abnormalities in the in vivo imaging (Figure 1C,D), highlighting limitations in SLO/OCT resolution. Although the changes at day 3 post‐injection in SLO/OCT and LM seemed moderate, ultrastructural analysis revealed severe alterations in the RPE cells, such as the loss of basal infoldings and microvilli, flattened or rounded morphology of most RPE cells and general disorganization of the RPE layer (Figure 3), similar to the ultrastructural changes of the RPE found in AMD patients [14, 15, 16] or in a NaIO_3_ mouse model [13].

Interestingly, pathological changes including swelling and mitochondrial damage were also observed in the endothelial cells of the CC (Figure 4B). Given that endothelial cells and pericytes are sensitive to oxidative stress [17, 18].

NaIO_3_‐induced oxidative stress seems to directly affect the endothelial cells of the CC vessels, a phenomenon not yet properly studied. Also, still no consensus exists on the processes responsible for CC loss in GA [19].

We observed regions with prominent CC loss both with and without overlying RPE, but no areas with intact CC lacking overlying RPE (Figure 3B). This aligns with findings in the eyes of older human donors, where significant CC loss occurred, although RPE and retina remained intact. The loss of CC was further increased in donors with AMD. The data implicate that CC breakdown already occurs during normal aging and probably precedes degeneration of the RPE and retina [16].

Our IHC analyses revealed a loss of RPE65 in the RPE and infiltration of Iba1^+^ cells throughout the retina, which are common early symptoms in GA patients [20, 21, 22].

At this early time point only a few CD68^+^cells were present at the CC/RPE interface. Additionally, in the same region intermediate activation of GFAP and vimentin, an increased accumulation of the complement factors C3 and C5b‐9 along with a reduction of FB were observed.

As indicated in Table 1, by day 7 post‐injection using the aforementioned methods all typical changes demonstrated in patients with GA were also observed in our model, making this time point particularly suitable for evaluating the effects of potential treatments.

**TABLE 1:** NaIO_3_‐induced changes in our model 7 days after injection, detected using the indicated methods and the corresponding pathological changes observed in GA patients using the same methods.

	The present rat NaIO_3_ model 7 days post NaIO_3_ injection	Patients with GA
SLO	Hyper and hypo‐autofluorescent flecks (Figure 1A)	Diffuse‐trickling fundus auto‐fluorescence pattern [23, 24]
OCT	Wavy changes in the RPE reflection area (Figure 1B) and reduction of the retinal thickness (Figure S2)	Wavy hyperreflective foci at RPE‐Bruch‘s membrane reflection area [20, 23]
LM	ONL undulation, RPE migration, agglomeration and regional loss, loss of photoreceptors (PR) (Figures 1C,D and 2 respectively)	ONL undulation [25] PR and RPE regional loss, RPE migration, various stages of CC and RPE degeneration [16, 20, 26]
EM	Gradual degeneration of CC, RPE, PR; remodeling of ECM of Bruch‘s membrane, basal deposits, phagocytic cells invasion, granulocytes (Figures 3 and 4)	Degeneration of RPE and PR, CC loss [16], invasion of phagocytic cells [16, 27, 28, 29]
IHC	Gradual loss of RPE65 signal, infiltration of Iba1^+^ and CD68^+^cells, gradual increase of GFAP and vimentin reactivity, gradual increase of C3 with peak on day 7, and C5b‐9 reactivity increase (Figures 5, 6, and S1)	RPE65 signal loss [20], infiltrating of Iba1^+^ [21, 22] and CD68^+^cells [20], presence of granulocytes [30], increase of GFAP and vimentin reactivity [31, 32], increase of C3 [27, 33] and C5b‐9 reactivity [27]

For instance, a diffuse‐trickling fundus auto‐fluorescence pattern in SLO is considered a hallmark of GA in patients [23, 24], a very similar pattern of hyper‐ and hypo‐autofluorescent flecks was observed in our model (Figure 1A). In GA patients, hyper‐reflective foci at the RPE‐Bruch's membrane reflection area in OCT have been described [20, 23] consistent with the changes in the RPE/Bruch's membrane reflection area in our model (Figure 1B). The changes observed using LM shown in Figure 1C such as RPE migration, accumulation, formation of multilayers, morphological changes and rosette formations have also been described in patients with GA [16, 20].

Our IHC results showed, the further remaining loss of RPE65 which started at day 3 post‐ injection and revealed a peak of immune system activity involving a continuing invasion of Iba1^+^ and an emerging infiltration of CD68^+^cells, activation of GFAP and vimentin, and changes in the complement system such as reduced FB and increased C3 and C5b‐9 reactivity (Figures 5 and 6). Interestingly, the infiltration area of Iba1^+^ cells was all over the retina, whereas CD68^+^ cells were located exclusively in the CC/RPE area (Figure 5). For both Iba1^+^ [21, 22] and CD68^+^ cells [20], a similar invasion has been described in GA patients, as well as an activation of GFAP and vimentin [31, 32] and a reduced FB‐ and increased C3 and C5b‐9 reactivity [27, 33] have also been found in GA patients.

The most prominent ultrastructural changes at this time point were pronounced degenerative changes of RPE cells, patchy loss of CC, basal deposits on and structural changes of the ECM of the Bruch's membrane; also the appearance of numerous phagocytic cells—microglia and macrophages (Figure 3C), All these features have also been found in patients with GA [16, 27, 28, 29, 30].

Interestingly we also detected granulocytes in CC vessels as shown in Figure 3C2. Granulocytes are the effector cells of complement activation [34]. There is a growing body of evidence that an accumulation of mast cells (a type of granulocytes)—attracted and activated by complement‐mediated anaphylatoxins—plays an important role in AMD [35, 36, 37, 38]. Additionally, granulocyte infiltration into the retina was observed using IHC in patients with AMD and in a mouse model with an AMD‐like phenotype [30]. Moreover, an elevated expression of granulocyte colony‐stimulating factor (G‐CSF) was found in RPE/choroid macular punches from AMD donors [39]. Unfortunately to our knowledge, there are no EM‐based analyses on this subject. In our model granulocytes were detected exclusively at 7 days post NaIO_3_ injection.

At 14 days post‐injection, our SLO/OCT results and LM images showed slightly stronger but similar changes as at 7 days post‐injection (Figure 1A,B respectively) and a significant retinal thinning in OCT analyses (Figure S2) which correlates with the photoreceptor loss measured in LM images (Figure 2C). Ultrastructural analysis revealed similar but more pronounced changes across all tissues of the CC‐RPE complex compared to day 7, a slightly reduced number of phagocytic cells and absence of granulocytes. IHC showed reduced C3 accumulation compared to day 7 and the lowest CB reactivity in the observation period (Figure 6). Therefore, the time point of 7 days post NaIO_3_ injection is favorable for testing potential treatment approaches, as it exhibits a more pronounced activity of the immune system.

Another important finding is shown in Figure S1. The reactivity for FH did not change throughout the entire observation period in our model, which correlates with the situation observed in GA patients, where the immunoreactivity score for FH was not significantly different compared to that of aged controls [40]. Interestingly, in a mouse NaIO_3_ model an increase of FH was observed [41] again suggesting a stronger similarity between the complement system of rats and humans compared to mice. Although FB and C5 are considered targets for treatment approaches for GA [2, 42], to our knowledge there are no comprehensive studies analyzing their accumulation in GA patients or NaIO_3_ animal models which could be compared to data presented in the present study.

Additionally, due to their important role, we explicitly focused our EM analysis on mitochondria in RPE cells. Mitochondrial damage in the RPE has been implicated in the pathophysiology of several age‐related diseases including AMD. EM studies demonstrated that aged individuals have fewer and smaller mitochondria in the RPE as well as pathological ultrastructural changes such as loss of cristae and matrix density [43]. The reduction in number and structural changes is even more severe in patients with AMD, where mitochondria show bleb formation or interruption of their internal and external membranes [15]. Mitochondrial damage in the RPE, with ultrastructural changes similar to those seen in AMD patients and decreased mitochondria numbers, after NaIO_3_ treatment have been shown in mice [44] and rats [45]. Moreover, mitochondrial damage and dysfunction were demonstrated to be early effects induced by NaIO_3_ [46]. In our study we also observed severe mitochondrial damage in the RPE (Figure 4A). As shown in Figure 4C, 3 days after NaIO_3_injection, more than 70% of the mitochondria were already damaged; at 7 and 14 days post‐injection, over 90% were affected.

The degeneration of RPE cells and photoreceptor OS as indicated in Figure 1 and Figure 3 suggested, that the retinal function should be impaired by NaIO_3_. On the other hand, surviving RPE cells and photoreceptors were present throughout the whole observation period. To pursue the question of how strongly NaIO_3_ treatment influences the retinal function, ERG analyses were performed.

A drastic decrease in ERG responses following NaIO_3_ treatment has been observed in both rats [11, 44, 47, 48, 49] and mice [10, 47, 50, 51]. As expected, in our experiment, NaIO_3_ administration resulted in no ERG readings for low light intensities and only nearly flat ERG responses even for the high light intensity stimuli from day 3 onwards (Figure 7).

The exact mechanisms leading to the reduction of a‐ and b‐waves remain unknown, but several potential mechanisms have been proposed. For instance NaIO_3_ dramatically induces reactive oxygen species (ROS) production leading to oxidative stress in the RPE [52] and also directly in the photoreceptors altering their function and survival [53]. Oxidative stress further contributes to blood‐retina barrier (BRB) damage by inducing the loss of RPE cell–cell connections [54], disruption of tight junctions, changing the composition of the ECM of the Bruch's membrane and subsequent BRB breakdown [10, 55, 56], which exposes photoreceptors to harmful substances from the choroidal circulation, accelerating retinal degeneration. In patients age‐related or pathological changes of the BRB are associated with reduced ERG readings [54, 57]. Given that NaIO_3_‐induced RPE cell death occurs primarily through necrosis, necroptosis, ferroptosis, and pyroptosis, and only partly through apoptosis [5]. These processes lead to the release of ions, particularly iron, which interferes with the generation of electrical signals in the OS [58]. Another highly important effector molecule affected by NaIO_3_‐treatment is RPE65. RPE65 plays an essential role in the visual cycle; its absence leads to flat ERG readings [59].

As indicated in Figure 5A in our model the RPE cells have lost their ability to express RPE65 from day 3 post NaIO_3_ injection onwards, which may also explain the reduction of the ERG signals.

In conclusion, comprehensive monitoring using a broad variety of techniques is crucial to fully understand NaIO_3_‐induced changes. Our model faithfully recapitulates key GA features and represents a useful tool to evaluate novel therapies aimed at protecting the choriocapillaris and RPE to prevent or slow down GA. For analyses of such therapy approaches in our model, day 7 post NaIO_3_ injection has shown itself to be the most suitable time point of analysis, as it exhibits pronounced GA‐like changes, a peak of phagocytic and immune cell invasion and a peak of C3 and C5b‐9 accumulation in the CC/RPE interface area.

## Author Contributions

S.Z., S.J.C., A.V.T. and S.J. wrote the manuscript. S.Z., A.V.T. and S.J. conducted the experiments. S.Z., F.V., J.T. and A.V.T. acquired and analyzed the data. S.J. designed the study. All authors were involved in drafting and revising the manuscript.

## Funding

This work was supported by Dr. Werner Jackstaedt Stiftung, Germany, Guangzhou Elites Scholarship Council, China, and Open access Publishing Fund of University of Tuebingen.

## Conflicts of Interest

The authors declare no conflicts of interest.

## Supporting information


**Figure S1:** C5 and FH immunoreactivities in red, nuclei were counterstained with DAPI (blue) and autofluorescence (AF) appeared in greenish yellow. No specific signal changes could be detected at any of the time points.

**Figure S2:** Statistical analysis of the mean retinal thickness for the different groups measured on OCT images from cross sections of the central part of the eye: 0 days (pretreatment), 3, 7 and 14 days after NaIO_3_ treatment. *n* = 6 eyes per group, ANOVA with Dunn‘s posttest, *p < 0.05, ***p < 0.0001.

## Data Availability

The data that support the findings of this study are available in the methods of this article.
