# Supplementary figures and images for: Time Course of Structural, Functional, Complement Changes and Inflammatory Processes in a Sodium Iodate Rat Model of Geographic Atrophy
Source: FASEB J. 2025 Dec 4;39(23):e71307. doi: 10.1096/fj.202502226R (PMC12677118; doi:10.1096/fj.202502226R)

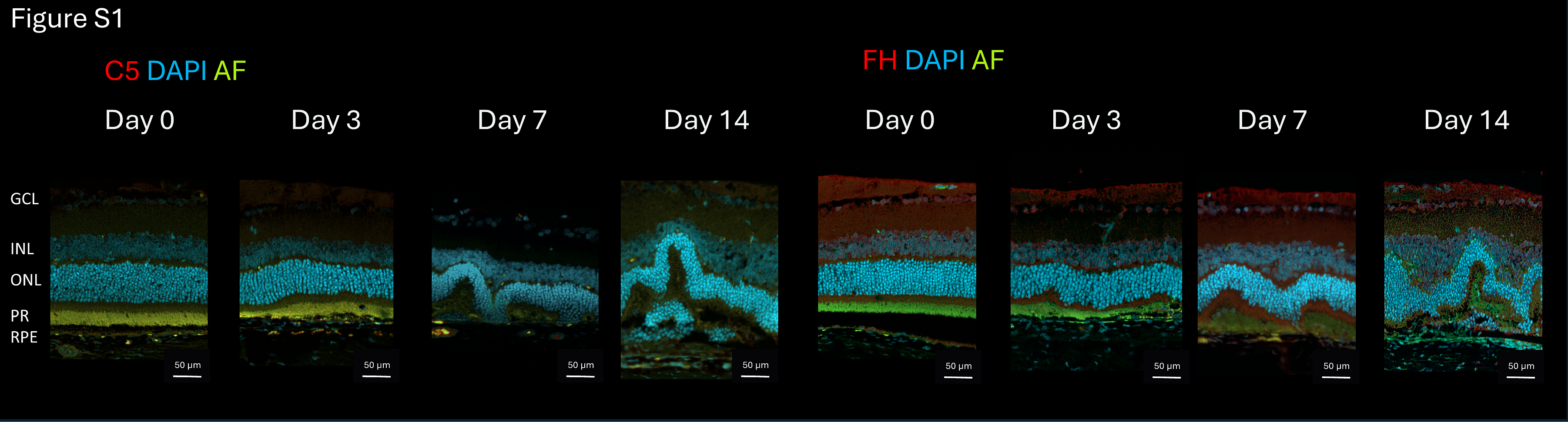

Supplement: Supplementary file 1 — Figure S1: C5 and FH immunoreactivities in red, nuclei were counterstained with DAPI (blue) and autofluorescence (AF) appeared in greenish yellow. No specific signal changes could be detected at any of the time points. [file FSB2-39-e71307-s002.tif]

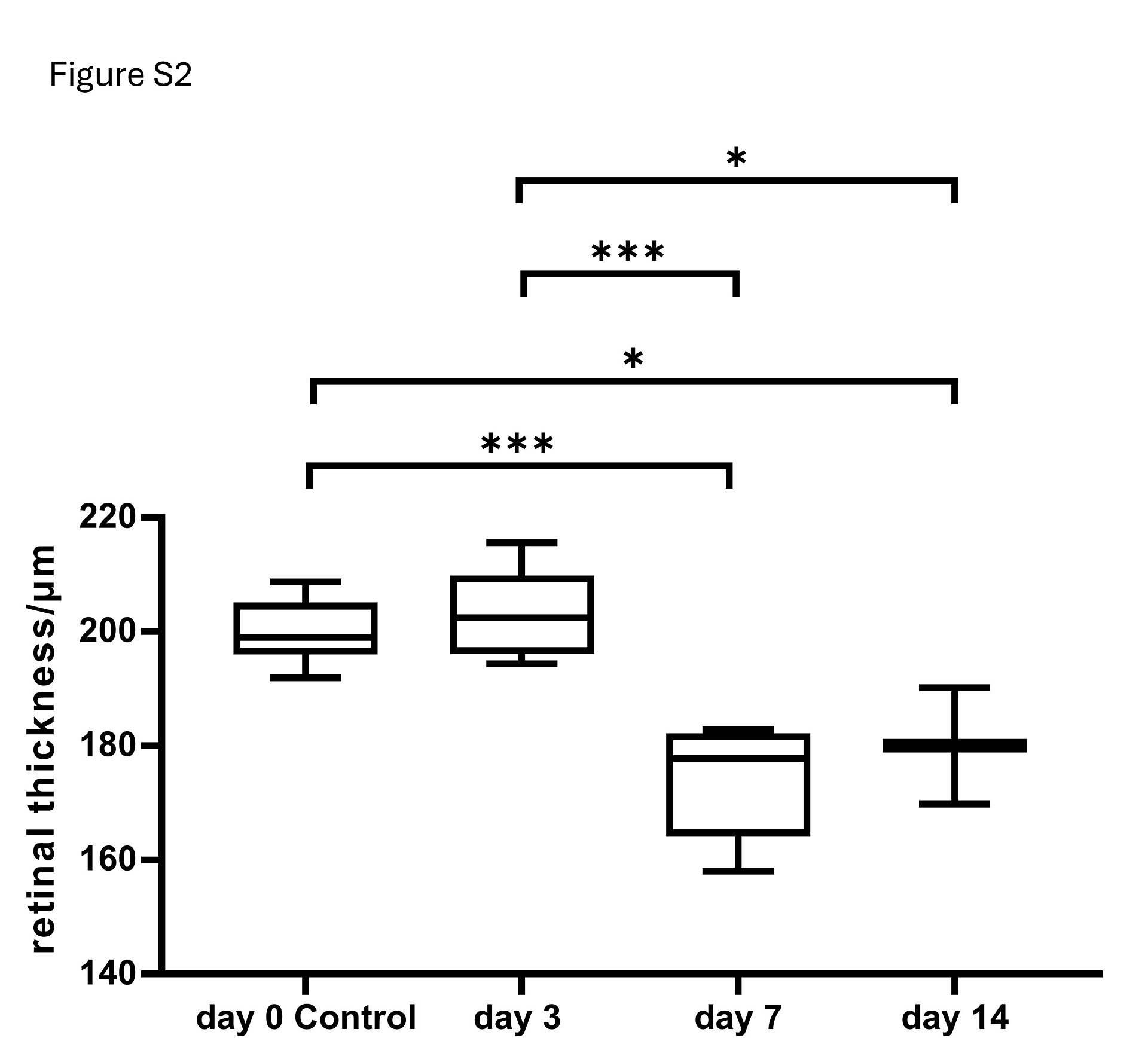

Supplement: Supplementary file 2 — Figure S2: Statistical analysis of the mean retinal thickness for the different groups measured on OCT images from cross sections of the central part of the eye: 0 days (pretreatment), 3, 7 and 14 days after NaIO3 treatment. n = 6 eyes per group, ANOVA with Dunn‘s posttest, *p < 0.05, ***p < 0.0001. [file FSB2-39-e71307-s001.tif]
